# Social support and coping in adults with type 2 diabetes

**DOI:** 10.4102/phcfm.v9i1.1405

**Published:** 2017-07-31

**Authors:** Samantha Ramkisson, Basil J. Pillay, Wilbert Sibanda

**Affiliations:** 1 Department of Behavioural Medicine, School of Nursing and Public Health, College of Health Sciences, University of KwaZulu-Natal, South Africa

## Abstract

**Background:**

The diagnosis of diabetes has been described as a lifelong psychological burden on the patient and his or her family. Social support plays a pivotal role in patients with diabetes and is important in enabling them to cope effectively with the disease. There is a dearth of research on social support and coping in patients with diabetes in South Africa.

**Objectives:**

The aim of this study was to explore whether patients with poor perceived social support have lower levels of well-being and coping than patients with good social support.

**Method:**

A cross-sectional study was conducted at both public and private facilities on the north coast of KwaZulu-Natal, South Africa. The Diabetes Care Profile (DCP), the General Health Questionnaire (GHQ), the Social Support Questionnaire (SSQ) and the WHO-5 Well-being Index (WHO-5) were administered to 401 participants.

**Results:**

The findings indicate that there is an inverse relationship between social support and coping, which suggests that an increase in social support is associated with a decrease in emotional distress. There was a small positive correlation between the SSQ and the WHO-5, which suggests participants who had good support endorsed better levels of well-being. Although participants indicated that they were satisfied with their level of support, they had poor coping as indicated by the high mean score on the GHQ and the high HbA1c level. There was a small positive correlation between GHQ and HbA1c. There was no association between social support and HbA1c.

**Conclusion:**

Social support is important in helping the patient with diabetes cope with the disease and to improve adherence to treatment. Health care providers should take cognisance of psychosocial factors in the treatment regime of the patient. Family members should be educated about diabetes, the importance of adherence and long-term complications of the disease.

## Background

The diagnosis of diabetes has been described as a lifelong psychological burden on the person and his or her family.^1^ Diabetes is a chronic, demanding disease, which requires daily management and strict adherence to medication, diet and exercise. Failure to adhere to these regimens can lead to uncontrolled blood sugar levels and complications such as blindness, kidney failure, leg amputation and nerve damage.^2^ Literature shows that social support plays a pivotal role in patients with diabetes and can contribute to the successful management of the disease.^3,4,5,6,7^ Social support helps with diabetes control and provides support for the necessary physical and dietary changes.^8^ Family support has been identified as the main source of support^9^ and plays an essential role in lifestyle changes and diabetes management.^6^ Because of the chronic nature of diabetes, patients make regular visits to health care providers and this can result in these health care providers also becoming part of their social support network.^8,9^

According to Grey,^10^ coping serves as a protective function that can eliminate or modify stressful conditions. The chronic disease burden of diabetes is considered to be a stressor with which the patient with diabetes is expected to cope with on a daily basis.^10,11^ Psychological and physical health depend on the ability of the patient to thoughtfully plan and adhere to routines of diet, activity and medication regardless of competing life demands, life stressors or temporary moods.^12^ Some patients are successful in integrating these demands of the disease into their daily lives; however, some patients have difficulty with accepting the disease, which leads to the inability to cope with it.^12,13^ This may lead to a decrease in the patient’s self-care, adherence to medication and have an adverse impact on metabolic control.^13^

In South Africa, 2.3 million people are estimated to be living with diabetes, with 1.39 million people who have not yet been diagnosed.^14^ In 2014, diabetes was among the top three leading causes of death in South Africa and the second leading cause of death in women.^15^

Despite the abundance of international research on social support and coping in patients with diabetes, there is a dearth of such research in South Africa. The aim of this study was to explore whether patients with poor perceived social support have lower levels of well-being and coping than patients with good social support locally.

## Method

Participants were recruited from two public health facilities and five private medical practices on the north coast of KwaZulu-Natal. The method used in this explorative study is similar to that used in other studies.^16,17^

### Participants

Patients who were 18 years and older had a diagnosis of type 2 diabetes for more than six months and who were able to speak English or isiZulu fluently were invited to participate in the study. The total sample consisted of 401 participants, 200 from the private health sector and 201 from the public health sector.

### Procedure

Trained research assistants who were fluent in both English and isiZulu explained the study to patients who were awaiting their regular appointments. Participation was voluntary, and those who met the inclusion criteria were invited to complete the questionnaires. After patients signed informed consent forms, questionnaires were administered to the participants in the language of their choice.

### Instruments

A questionnaire comprising a demographic section and four instruments was completed by the participants. Participants who had difficulty completing the questionnaires were helped by the trained research assistants. The average time to complete the questionnaires was 45–60 min.

The Diabetes Care Profile (DCP), developed by the Michigan Diabetes and Research Centre,^18^ is a self-administered questionnaire that assesses the social and psychological factors related to diabetes and its treatment. The instrument contains 234 items arranged in 16 scales. These scales assess the patients’ diabetes attitudes, diabetes beliefs, self-reported diabetes self-care and difficulties with diabetes self-care. The DCP also contains questions concerning demographic information and self-care practices. Cronbach’s alphas ranged from 0.60 to 0.95.^18^ The results of studies conducted by Fitzgerald and Davis^18^ and Fitzgerald and Anderson^19^ found the DCP to be a reliable and valid instrument for measuring the psychosocial factors related to diabetes and its treatment. The DCP-Support Scale was used in this study.

The General Health Questionnaire (GHQ-28), developed by Goldberg, is a 28-item measure of emotional distress.^20^ There are four subscales with the 28 items distributed evenly across each. The four subscales are somatic symptoms, anxiety and insomnia, social dysfunction and severe depression. Internal reliability ranges from 0.78 to 0.95;^21^ Cronbach’s alphas ranged from 0.86 to 0.92 in this study. The GHQ was used as a measure of coping in this study with high scores indicating emotional distress and poor coping.

The Social Support Questionnaire (SSQ), developed by Pillay,^22^ has 15 items, which measure the qualitative and quantitative aspects of a person’s network of relationships that help him or her in adverse circumstances. The SSQ is a bilingual instrument in English and isiZulu. The instrument consists of two parts. Participants are required to list people who provide support and then rate their level of satisfaction with the support on a four-point Likert scale as either very dissatisfied, dissatisfied, satisfied or very satisfied.^22^ The Cronbach’s alpha value in Pillay’s study was 0.91, while the Cronbach’s alpha value in this study was 0.95.

The WHO-5 has five items and covers positive mood (good spirits; relaxation), vitality (being active; waking up fresh and rested) and general interests (being interested in things).^23^ The degree to which these feelings were present in the previous two weeks is scored on a six-point Likert scale ranging from 0 (not present) to 5 (constantly present). The raw scores are transformed to a scaled score ranging from 0 to 100. A score of less than 52 suggests poor emotional well-being and is a sign for further testing. A score of 28 or less is indicative of depression. The Cronbach’s alpha of the WHO-5 in this study was 0.89.

Where necessary, the questionnaires were translated into isiZulu by an accredited isiZulu translator and were back-translated into English by an isiZulu educator.

### Data analysis

All analyses were performed using SPSS version 24.^24^ Continuous variables were summarised as means (*M*) and standard deviations (s.d.). Medians and interquartile ranges were used for variables that were highly skewed or had significant outliers. Categorical and ordinal variables were summarised using proportions and percentages. Proportions were compared using Pearson’s chi-square test or Fisher’s exact test, as appropriate. Pearson’s correlation coefficient was used to measure the linear dependence between WHO-5 and SSQ scales. Canonical correlation analysis (CCA) was used to examine the relationship between linear combinations of SSQ variables and linear combinations of GHQ variables.

### Ethical consideration

The Biomedical Ethical Research Committee of the University of KwaZulu-Natal granted ethics approval. Consent was obtained from the managers of the public facilities and the Provincial Department of Health. Research assistants explained to patients that participation was voluntary and had no bearing on their treatment.

## Results

Table 1 shows the demographic characteristics of the sample. More than a quarter of the sample were unemployed 114 (28.43%), while 304 (75.81%) had an educational level of below Grade 12. A majority of the patients were female (243; 60.60%), with 276 (68.80%) reporting that they were married and 106 (96.90%) indicating that they lived with five or more people. In terms of ethnic groups, 100 (24.90%) were Black African, three (0.75%) were mixed race, 274 (68.33%) were Indian and 24 (6.00%) were White. There were 200 (49.88%) patients from the private sector and 201 (50.12%) patients from the public sector.

**TABLE 1:** Demographic characteristics of the total study sample.

Characteristic	*n*	%
Educational level		
< Grade 12	304	75.81
Post Grade 12	97	24.19
Employment		
Employed	183	45.64
Retired or homemaker	104	25.94
Unemployed	114	28.43
Gender		
Female	243	60.60
Male	158	39.40
Marital status		
Married	276	68.80
Never married	53	13.20
Separated/divorced	24	6.00
Widowed	48	12.00
Number of people who live with you		
Live alone	18	4.3
1 Person	57	13.8
2 People	74	17.9
3 People	77	18.6
4 People	69	16.7
Five or more people	106	96.9
Race		
Black African	100	24.90
Mixed race	3	0.75
Indian	274	68.33
White	24	6.00
Sector		
Private	200	49.88
Public	201	50.12

The mean age of the study sample was 53.70 years (s.d. = 10.7). The average HbA1c^a^ was 12.02% (s.d. = 5.00), which is higher than the generally accepted norm of 7% or lower.^1^ The average duration of the disease for the sample was 10.3 years (s.d. = 7.9).

The level of satisfaction with social support as measured by the SSQ is shown in Table 2. Most of the participants were very satisfied with the level of support that they received. Most of the participants (327; 82.40%) endorsed the item that they were very satisfied with the person who truly loves them and with the support that they receive when they are ill (310; 78.50%). The means and standard deviations of the SSQ are presented in Table 3.

**TABLE 2:** Level of satisfaction on the Social Support Questionnaire.

Variable	Very dissatisfied	Dissatisfied	Satisfied	Very satisfied
	*n* (%)	*n* (%)	*n* (%)	*n* (%)
1. Who can you count on to listen to you when you need to talk?	2 (5.0)	4 (1.0)	100 (25.0)	294 (73.5)
2. Who could you really count on to help in an emergency?	1 (0.3)	5 (1.3)	86 (21.6)	307 (76.9)
3. Who helps you feel that you are a good and worthwhile person?	2 (0.5)	6 (1.5)	84 (21.0)	308 (77.0)
4. Who can you really go to when you are worried or under pressure?	3 (0.8)	5 (1.3)	94 (23.6)	297 (74.4)
5. Who can you count on when you generally need help for reasons other than an emergency?	2 (0.5)	3 (0.8)	92 (23.2)	299 (75.5)
6. Who can you really count on when things go wrong, you have a mishap or run out of luck?	1 (0.5)	3 (0.8)	90 (22.7)	302 (76.1)
7. Who can you really count on to give advice, guidance or useful suggestions that would help you to avoid mistakes?	0	6 (1.5)	96 (24.2)	295 (74.3)
8. Who do you feel will help if a family member or someone very close to you died?	4 (1.0)	4 (1.0)	80 (20.3)	306 (77.7)
9. Who do you feel truly loves you deeply?	1 (0.3)	6 (1.5)	63 (15.9)	327 (82.4)
10. Who can you count on to comfort or console you when you are very upset?	3 (0.8)	6 (1.5)	80 (20.2)	307 (77.5)
11. Who can you really count on to support you in major decisions or plans you make?	2 (0.5)	7 (1.8)	90 (22.7)	297 (75.0)
12. Who would you trust with a secret or information that could get you into trouble?	3 (0.8)	6 (1.5)	87 (22.1)	297 (75.6)
13. Who do you count on when you are ill?	1 (0.3)	4 (1.3)	80 (20.3)	310 (78.5)
14. Who can you really count on when you get into trouble?	1 (0.3)	3 (0.8)	95 (23.9)	298 (75.1)
15. Who do you think will come to you if they had a need or problem?	2 (0.5)	4 (1.0)	83 (21.4)	299 (77.1)

**TABLE 3:** Means and standard deviations of the Social Support Questionnaire items.

Item	Mean (s.d.)
1. Who can you count on to listen to you when you need to talk?	3.75 (0.50)
2. Who could you really count on to help in an emergency?	3.75 (0.48)
3. Who helps you feel that you are a good and worthwhile person?	3.75 (0.50)
4. Who can you really go to when you are worried or under pressure?	3.72 (0.52)
5. Who can you count on when you generally need help for reasons other than an emergency?	3.74 (0.49)
6. Who can you really count on when things go wrong, you have a mishap or run out of luck?	3.74 (0.49)
7. Who can you really count on to give advice, guidance or useful suggestions that would help you to avoid mistakes?	3.73 (0.48)
8. Who do you feel will help if a family member or someone very close to you died?	3.75 (0.52)
9. Who do you feel truly loves you deeply?	3.80 (0.45)
10. Who can you count on to comfort or console you when you are very upset?	3.74 (0.52)
11. Who can you really count on to support you in major decisions or plans you make?	3.72 (0.52)
12. Who would you trust with a secret or information that could get you into trouble?	3.73 (0.53)
13. Who do you count on when you are ill?	3.80 (0.46)
14. Who can you really count on when you get into trouble?	3.74 (0.47)
15. Who do you think will come to you if they had a need or problem?	3.75 (0.50)

s.d., standard deviation.

Patients’ social support from family and friends as measured by the DCP is shown in Table 4. Just under half of the patients felt that they wanted a lot of help and support in following a meal plan (189; 47.10%), taking medicine (187; 46.63%), taking care of their feet (196; 48.88%), getting enough physical exercise (182; 45.39%) and testing sugar levels (183; 45.64%). Half of the patients (202; 50.37%) indicated that they want a lot of help and support in handling their feelings about diabetes. Most of the patients indicated that family and friends do help and support them in the abovementioned activities and (191; 47.63%) received help and support in handling their feelings about diabetes. Most of the patients felt that their family and friends accepted them as a patient with diabetes (315; 78.55%), received encouragement about living with diabetes (257; 64.09%) and felt that their family and friends listened to them when they wanted to talk about diabetes (242; 60.35%). A small percentage (50; 12.47%) felt that their family or friends nagged them about diabetes.

**TABLE 4:** Social support from family and friends on the Diabetes Care Profile.

Variable	Strongly disagree	Somewhat disagree	Neutral	Somewhat agree	Strongly agree	Does not apply
	*n* (%)	*n* (%)	*n* (%)	*n* (%)	*n* (%)	*n* (%)
I want a lot of help and support from my family or friends in:						
a) Following a meal plan	43 (10.72)	35 (8.73)	51 (12.72)	82 (20.45)	189 (47.13)	1 (0.25)
b) Taking my medicine	78 (19.45)	49 (12.22)	29 (7.23)	55 (13.72)	187 (46.63)	3 (0.75)
c) Taking care of my feet	65 (16.21)	36 (8.98)	36 (8.98)	64 (15.96)	196 (48.88)	4 (1.00)
d) Getting enough physical exercise	57 (14.21)	42 (10.47)	39 (9.73)ç	79 (19.70)	182 (45.39)	2 (0.50)
e) Testing my sugar	74 (18.45)	40 (9.98)	38 (9.48)	61 (15.21)	183 (45.64)	5 (1.25)
f) Handling my feelings about diabetes	52 (12.97)	25 (6.23)	41 (10.22)	80 (19.95)	202 (50.37)	1 (0.25)
My family or friends help and support me a lot to:						
a) Following a meal plan	57 (14.21)	40 (9.98)	34 (8.48)	92 (22.94)	178 (44.39)	-
b) Taking my medicine	82 (20.45)	41 (10.22)	24 (5.99)	65 (16.21)	186 (46.38)	3 (0.75)
c) Taking care of my feet	89 (22.19)	49 (12.22)	28 (6.98)	71 (17.71)	159 (39.65)	5 (1.25)
d) Getting enough physical exercise	81 (20.20)	47 (11.72)	40 (9.98)	88 (21.95)	145 (36.16)	-
e) Testing my sugar level	98 (24.44)	37 (9.23)	29 (7.23)	62 (15.46)	168 (41.90)	7 (1.75)
f) Handling my feelings about diabetes	51 (12.72)	34 (8.48)	36 (8.98)	87 (21.70)	191 (47.63)	2 (0.50
My family or friends:						
a) Accept me and my diabetes	3 (0.75)	3 (0.75)	14 (3.49)	66 (16.46)	315 (78.55)	_
b) Feel uncomfortable about me and my diabetes	288 (71.82)	54 (13.47)	25 (6.23)	21 (5.24)	13 (3.24)	_
c) Encourage or reassure me about my diabetes	19 (4.74)	11 (2.74)	34 (8.48)	80 (18.95)	257 (64.09)	_
d) Discourage or upset me about my diabetes	307 (76.56)	45 (11.22)	30 (7.48)	13 (3.24)	6 (1.50)	_
e) Listen to me when I want to talk about my diabetes	13 (3.24)	12 (2.99)	41 (10.22)	93 (23.19)	242 (60.35)	_
f) Nag me about diabetes	235 (58.60)	35 (8.73)	40 (9.98)	41 (10.22)	50 (12.47)	_

On the SSQ, the majority of the patients endorsed their spouse as providing the main social support. The DCP yielded similar results with patients indicating that most help in caring for their diabetes came from their spouse (197; 49.13%) and other family members (121; 30.17%). Thirty-seven patients (9.23%) indicated that the doctor helped most with diabetes.

Mean scores comparisons of the SSQ and GHQ by gender and health sectors are displayed in Table 5. The difference in mean scores between women (*M* = 55.86; s.d. = 6.70) and men (*M* = 54.87; s.d. = 8.32) was not statistically significant. There was also no statistically significant difference between the public and private health care sectors with mean scores of (*M* = 55.64; s.d. = 6.60) and (*M* = 55.31; s.d. = 8.10), respectively. On the GHQ, 159 (40%) participants reported elevated somatic symptoms, 149 (37.16%) had high levels of anxiety, 290 (72.32%) reported high levels of social dysfunction and 78 (19%) had severe depressive symptoms. Women (*M* = 25.50; s.d. = 16.11) had significantly higher mean GHQ scores compared with men (*M* = 18.40; s.d. = 12.80). The public sector mean GHQ score (*M* = 25.20; s.d. = 15.84) was significantly higher than that of the private sector (*M* = 20.16; s.d. = 14.30). The total GHQ mean score (*M* = 22.70; s.d. = 15.30) was elevated, indicating distress and difficulty in coping.

**TABLE 5:** Comparison of the Social Support Questionnaire and General Health Questionnaire mean scores for gender and health sector.

Variable	Gender					Sector						
	Male M (s.d.)	95% CI	Female M (s.d.)	95% CI	*p*-value*	Public M (s.d.)	95% CI	Private M (s.d.)	95% CI	*p*-value	Total M (s.d.)	95% CI
SSQ	54.87 (8.32)	(53.60 – 56.20)	55.86 (6.70)	(55.02 – 56.71)	0.193	55.64 (6.60)	(54.72 – 56.55)	55.31 (8.10)	(54.18 – 56.44)	0.658	55.47 (7.38)	(54.74 – 56.20)
GHQ												
Somatic	11.75 (3.91)	(11.13 – 12.40)	14.43 (4.95)	(13.80 – 15.10)	< 0.001	14. 10 (4.80)	(13.40 – 14.74)	12.70 (4.61)	(12.03 – 13.32)	0.003	13.37 (4.75)	(12.91 – 13.84)
Anxiety Insomnia	11.50 (4.51)	(10.77 – 12.20)	13.54 (5.20)	(12.88 – 14.20)	< 0.001	13.30 (5.13)	(12.60 – 14.01)	12.20 (4.87)	(11.50 – 12.83)	0.022	12.73 (5.03)	(12.24 – 13.22]
Social Dysfunction	14.15 (3.12)	(13.65 – 14.63)	15.17 (3.69)	(14.71 – 15.64)	0.022	15.44 (3.90)	(14.90 – 16.00)	14.09 (2.92)	(13.68 – 14.50)	< 0.001	14.77 (3.51)	(14.43 – 15.11)
Depression	9.01 (3.70)	(8.43 – 9.60)	10.30 (5.03)	(9.70 – 10.94)	0.006	10.35 (4.8)	(9.70 – 11.01)	9.24 (4.33)	(8.64 – 9.84)	0.016	9.80 (4.60)	(9.34 – 10.25)
Total	18.40 (12.80)	(16.38 – 20.40)	25.50 (16.11)	(23.41 – 27.50)	< 0.001	25.20 (15.84)	(23.00 – 27.40)	20.16 (14.30)	(18.17 – 22.14)	0.001	22.70 (15.30)	(21.80 – 24.20)

CI, confidence interval; GHQ, General Health Questionnaire; s.d., standard deviation; SSQ, Social Support Questionnaire.

* Chi-squared (*χ*^2^) test or Fisher’s exact test (if expected cell count < 5) used to compare categorical variable.

A canonical correlation was used to identify and measure the associations between multiple SSQ and GHQ variables. The null hypothesis of the canonical correlation was that there is no linear correlation between the support (SSQ) and coping (GHQ) variables. The null hypothesis that social support variables are independent from the coping variables is tested using Wilks’s lambda. For this study, the Wilks’s lambda results were: *Λ* = 0.78759; *F* = 1.46332; df = 60; *p* < 0.013. In general, Wilks’s lambda is the opposite of the *R*-squared, where a near zero indicates that there was a significant difference between SSQ and the GHQ (*p* < 0.014), which suggests that there is a significant relationship between social support and coping. The null hypothesis that there is no relationship between the two sets of variables is rejected; therefore, it can be concluded that the two sets of variables are dependent. The Pearson product-moment correlation coefficient was used to measure the strength of linear association between individual SSQ (social support) and GHQ (coping) variables. There was a small negative correlation between the two variables (*r* = -0.099, *N* = 401, *p* < 0.05), with high levels of support associated with a decrease in coping.

A univariate analysis of variance was used, to explore which support items were significant and had a significant effect on coping. The following items were significant: ‘Who can you count on to listen to you when you need to talk?’ (*p* < 0.025); ‘Who can you really count on to give advice, guidance or useful suggestions that would help you to avoid mistakes?’ (*p* < 0.004), ‘Who do you feel truly loves you deeply?’ (*p* < 0.030), ‘Who can you count on to comfort or console you when you are very upset?’ (*p* < 0.003) and ‘Who can you really count on when you get into trouble?’ (*p* < 0.033).

The relationship between the WHO-5 and the SSQ scales was investigated using Pearson’s correlation coefficient. There was a small statistically significant (*p* < 0.01) positive correlation between the scales (*ρ* = +0.14) which suggests participants who had good support reported better levels of well-being. Three items on the SSQ showed statistical significance (*p* < 0.05). These items were: ‘Who could you really count on to help in an emergency?’ (*p* < 0.036), ‘Who can you really count on to give advice, guidance or useful suggestions that would help you to avoid mistakes?’ (*p* < 0.035); and ‘Who do you feel will help if a family member or someone close to you died?’ (*p* < 0.037).

In terms of ethnic groups, the following results were found: there was a strong negative correlation between social support and coping in the White ethnic group (*r* = -0.58, *n* = 24, *p <* 0.01), with high levels of support associated with decreased symptoms of emotional distress (which indicates better coping). There was a small positive correlation between social support and coping in the Black ethnic group (*r* = 0.124, *n* = 100, *p* = 0.219). There was a medium negative correlation in the Indian ethnic group (*r* = -0.34, *n* = 273, *p* = 0.580). There was no statistical significance in either group.

The linear correlation between HbA1c and social support was not statistically significant (*r* = 0.051, *N* = 401, *p* < 0.335). This indicates that there is no association between glycaemic control and social support. There was a statistically significant, but small positive correlation between HbA1c and coping (*r* = 0.17, *N* = 401, *p* < 0.001), with an increase in HbA1c levels associated with an increase in emotional distress.

## Discussion

The findings indicate an inverse relationship between social support and coping, which suggests that an increase in social support is associated with a decrease in emotional distress. Participants who reported high levels of social support had better well-being levels. There was a small positive correlation between GHQ and HbA1c. Women and patients who attended public sector health facilities indicated poor coping.

The inverse relationship between social support and coping indicates that increased social support is associated with decreased emotional distress. Specific emotional support was associated with decreased emotional distress. Participants who endorsed high levels of social support had better levels of well-being. This is in keeping with a local study which had similar findings.^25^ Specific social support in terms of emergency situations was associated with better well-being levels.

Participants from both the private and the public sectors were satisfied with the level of support that they received. In this sample, most participants lived with five or more people, and the majority were married. The majority of patients indicated that most support came from their spouse or other family members. This is in agreement with the literature that shows that family support plays a vital role in the management of diabetes^6^ and can assist with the self-care behaviours of the patient.^26^ As noted by Litman,^27^ the family is the most important context in which illness can be resolved; therefore, social support from family can help in treatment outcomes for the patient. It is, therefore, important to include the family as part of the treatment regime of the patient. Families of patients with diabetes can also be referred to mental health care practitioners, who can facilitate support groups for families and help them cope with the needs of their family members with diabetes.

Most participants reported that they needed help with diet, medicine and exercise. Participants also indicated that they receive practical support, such as with diet and exercise, from family and friends. These findings were similar to a study conducted by Oftedal^5^ who found that patients viewed practical support as being helpful and valuable, which also stimulated effective diabetes management. Practical support and social support were found to be highly correlated with adherence.^22,28^ Adherence to diet, medication and exercise helps with glycaemic control. The treatment goal in diabetes is to achieve strict control of blood sugar levels,^29^ thereby delaying or preventing long-term complications. Uncontrolled blood sugar levels can lead to serious diseases affecting the heart and blood vessels, eyes, kidneys and nerves.^14^ Therefore, practical support received from family and friends in assisting with adherence to medication, diet and exercise forms an important part in the treatment goal for achieving good glycaemic control.

Although the White ethnic group sample number was small, they reported high levels of social support which was associated with decreased levels of emotional distress and better coping. Historically in South Africa, the White ethnic group had better access to specialised health care compared to other ethnic groups.^30,31^ There is still a disparity in health care services with most patients from the Black ethnic group accessing public health care facilities.^31^ Patients from previously disadvantaged groups often do not understand the importance of adherence to treatment and the severity of the potential long-term complications.^32^

In this study, both men and women indicated that they were satisfied with the level of support they received, which is contrary to other studies which found that women had lower levels of support compared with men.^33,34^ However, females had a high mean score on the GHQ, which indicates that women had greater difficulty coping with the disease. This is in keeping with other studies that found women tend to cope poorly with the disease.^33,35^ In South Africa, women are often the breadwinners in the home and have difficulty coping with the added demands of their chronic disease in addition to taking care of the needs of the family.^34,36^ Furthermore, because of low socioeconomic status and poor access to health care, women often have a lack of knowledge about the disease and its treatment,^36^ which impedes positive treatment outcomes.

There was no association between HbA1c and social support. Studies are not clear about social support and diabetes control. Some studies show that poor social support is strongly associated with poor diabetes control^11,28^ while others found that support was not associated with metabolic control.^25,37^ Although most of the participants were satisfied with their level of social support, the high HbA1c level of 12.02% indicates that patients in this sample are not coping well with the demands of the disease. Given that the generally accepted norm is 7% or lower,^1^ the high HbA1c level indicates poor management of the disease. Psychological stress with a helplessness reaction can lead to an activation of the hypothalamic-pituitary-adrenal axis, which results in high cortisol levels which in turn antagonise the actions of insulin.^38^ Support may reduce the perception that a situation is stressful thereby decreasing neuroendocrine responses to stress.^39^ Although family and friends are willing to render support, they may not know how to provide better and more effective support as reported in the DAWN2 study, where 37.1% of respondents were frustrated that they did not know how best to help the patient with diabetes but wanted to be more involved in their care.^40^ Therefore, family and friends need to be advised by health care providers as to how to render support that will positively impact treatment outcomes.^41^

Diabetes is a demanding disease, which involves lifestyle changes in terms of diet, exercise and adherence to medication. Patients can feel ‘burned out’ and overwhelmed by these demands,^42^ which lead to diabetes distress. In this study, there was a small positive correlation between HbA1c and the GHQ, which indicates that an increase in HbA1c levels is associated with an increase in emotional distress. High levels of HbA1c are associated with high levels of diabetes distress,^43,44^ which can lead to various complications. Often the psychological aspects of the disease, such as diabetes distress, are neglected which affects glycaemic control.^43,45^ In some patients, high diabetes distress can negatively influence their self-management and adherence to medication, with consequential effects on glycaemic control, while in other patients, poor glycaemic control can lead to distress and can influence disease management.^43^ In this regard, the family can play a role in assisting the patient by providing not only practical support but also emotional support. Patients and the family will also benefit from consultations with a mental health practitioner, as well as lay counsellors, lay support groups and faith-based groups to help them manage and cope with diabetes distress.

Patients from the public sector had a high mean score on the GHQ scale, which indicates poor coping. Having a chronic disease means attending health care services often; therefore, health care practitioners become a source of support and play a critical role in the management of diabetes.^46^ However, patients who utilise the state services are seen by different health care practitioners when they visit the hospital. A further concern is that, more often than not, the practitioners in the state services have extremely high service loads and lack of basic resources. Given these problems of not having a regular practitioner seeing patients, high work load and poor resources, practitioners do not have sufficient time to establish empathy and good doctor–patient relations, as shown in other research.^47^ Researchers have identified the lack of communication between patients and health care providers as an area of concern. However, empathy and building trust with patients are helpful to bring about behaviour change in patients.^48^ Health care providers, therefore, play an essential supportive role in the treatment of the patient with diabetes.^8,48^ Health care providers need to take cognisance of their supportive role in treating patients by adopting empathetic relationships with their patients and also increasing the level of communication, which will lead to greater social support for the patient.

The facts are reasonably well presented but there is little interrogation of the results including poor comparison of literature to either support or refute claims.

## Conclusion

This study supports the findings of the growing body of literature which emphasises the need for, and the importance of, social support in helping the patient with diabetes cope with the disease and to improve adherence to treatment. Health care providers need to take cognisance of psychosocial factors in the treatment regime of the patient. The mental health practitioner should form part of the multidisciplinary team involved in type 2 diabetes management to facilitate support groups for family members and provide interventions for both the patient with diabetes and their families. Policy guidelines should include the family as a part of the treatment plan for the patient with diabetes.

### Limitations

A limitation of this study is the cross-sectional design. Surveys and questionnaires carry a huge amount of research bias. While this was an exploratory study, future studies to investigate the link between social support and coping in diabetes should use more rigorous methods. Furthermore, some of the ethnic groups’ samples sizes were small and, therefore, results cannot be generalised.

## Footnotes

a HbA1C is an average measure of assessing glycaemic control over a period of three months (World Health Organisation, 2011).
